# The Penetration Ability of Calcium Silicate Root Canal Sealers into Dentinal Tubules Compared to Conventional Resin-Based Sealer: A Confocal Laser Scanning Microscopy Study

**DOI:** 10.3390/ma12030531

**Published:** 2019-02-11

**Authors:** Yemi Kim, Ban-Suk Kim, Yong-Min Kim, Donghee Lee, Sin-Young Kim

**Affiliations:** 1 Department of Conservative Dentistry, College of Medicine, Ewha Womans University, Seoul 07985, Korea; yemis@hanmail.net; 2 Department of Conservative Dentistry, Seoul St. Mary’s Dental Hospital, College of Medicine, The Catholic University of Korea, Seoul 06591, Korea; dentkim@catholic.ac.kr (B.-S.K.); yongmin32@nate.com (Y.-M.K.); 3 College of Medicine, The Catholic University of Korea, Seoul 06591, Korea; dong524@naver.com

**Keywords:** sealing ability, CLSM, calcium silicate sealer, resin-based conventional sealer

## Abstract

The purpose of this study was to compare the penetration ability of calcium silicate root canal sealers and conventional resin-based sealer using confocal laser scanning microscopy (CLSM). A total of 60 recently extracted single-rooted human premolars were used in this study. The root canals were prepared to a size 40/0.06 taper with ProFile rotary instruments and irrigated with NaOCl and EDTA. After drying all canals, the specimens were randomly divided into three experimental groups (*n* = 20): Group 1, gutta-percha (GP)/AH Plus with continuous wave compaction; group 2, GP/BioRoot RCS with a single-cone technique; and group 3, GP/Endoseal MTA with a single-cone technique. All experimental samples were sectioned perpendicular to their long axis using a low-speed diamond wheel at the apical, middle, and coronal third levels. The penetration abilities of all samples were evaluated using CLSM. A Kruskal–Wallis analysis and a series of Mann–Whitney *U* post hoc tests were performed. A higher intensity level was found in the coronal area and a lower intensity level in the apical area in all the experimental groups. The AH Plus group showed higher sum fluorescence intensity in the apical and coronal thirds compared with the BioRoot RCS and Endoseal MTA groups, whereas the BioRoot RCS group showed a higher intensity level in the middle third, similar to the AH Plus group. The maximum sealer penetration depth was low in the apical area and high in the coronal area in the AH Plus and Endoseal MTA groups. In the BioRoot RCS group, maximum sealer penetration was observed in the middle third. In conclusion, there were significant differences in sealer penetration pattern and distance according to the root level and sealer type.

## 1. Introduction

The main goal of root canal treatment is to provide three-dimensional obturation of the root canal system. A hermetic seal reduces coronal leakage and bacterial contamination, prevents apical periodontitis, and entombs the remaining irritants in the root canal [1,2,3]. Various endodontic materials have been developed for complete and impermeable fillings. Root canal sealers are necessary to seal the gap between the root dentin wall and the obturating material. Sealers should seal the root canal apically and laterally, and also fill voids and irregularities. The ability of the sealer to penetrate into the dentinal tubules is important, as this helps the sealer provide a fluid-tight seal [4,5] and prevent penetration by microorganisms and toxins [6].

Resin-based conventional root canal sealers have conventionally been used, offering the advantages of reduced solubility, tight apical sealing, and microretention to the root dentin. AH Plus (Dentsply DeTrey GmbH, Konstanz, Germany) is an epoxy resin-based sealer that is used in conjunction with gutta-percha (GP) in various root filling techniques. The toxicity of these sealers is reduced after setting; however, they exhibit toxicity when freshly mixed [7]. To overcome the problem of toxicity, new calcium silicate sealers have been developed.

BioRoot RCS (Septodont, St. Maur-des-Fossés, France) is composed mainly of tricalcium silicate and zirconium oxide powder that must be mixed with a liquid containing calcium chloride. In recent studies comparing epoxy resin-based and calcium silicate sealers, BioRoot RCS showed excellent biocompatibility in both the fresh and set states [7,8,9]. In another recent study, BioRoot RCS showed a higher void percentage compared with AH Plus [10]. 

Endoseal MTA (Maruchi, Wonju, Korea) is a pozzolan-based premixed calcium silicate sealer that offers satisfactory biocompatibility [11,12] and good root canal filling quality [13,14]. It has high alkalinity and low solubility, similar to ProRoot MTA (Dentsply Tulsa Dental Specialties, Johnson City, TN, USA) [15].

The purpose of this study was to compare the penetration ability of calcium silicate root canal sealers and conventional resin-based sealer using confocal laser scanning microscopy (CLSM). The null hypothesis of this study was as follows: There should be no difference in sealing ability among the different types of root canal sealers tested.

## 2. Materials and Methods

The protocol for this study was approved by the Institutional Review Board of Seoul St. Mary’s Dental Hospital, The Catholic University of Korea, Seoul, Korea (KC18SNSI0100). Written informed consent was obtained on the day the extraction was performed.

### 2.1. Preparation of Specimens

This study was approved by our institutional review board (IRB No. KC18SNSI0100). A total of 60 recently extracted single-rooted human premolars with intact coronal surfaces, which had been extracted for orthodontic treatment, were used in this study. The working length was determined at the point at which the #15 K-file was detected in the apex. The premolars were prepared using a crown-down technique to a size 40/0.06 taper with ProFile rotary instruments (Dentsply Maillefer, Ballaigues, Switzerland). The canals were then irrigated with 2 mL of 2.5% sodium hypochlorite (NaOCl) solution using a 27-gauge slotted, side-vented needle (Endo-Eze^®^ Tips; Ultradent Products, Inc., South Jordan, UT, USA) during the instrumentation procedure. After the preparation, the root canals were rinsed with 2 mL of 17% ethylenediaminetetraacetic acid (EDTA) solution for 1 min to remove the residual smear layer. As a final irrigation, 10 mL of distilled water was used.

### 2.2. Specimen Grouping and Root Canal Obturation

After drying all canals, the specimens were divided, according to the obturation technique and materials, into three experimental groups of 20 samples each, and the roots of all specimens were coated with two layers of nail varnish. The root canal filling materials tested in this study are listed in Table 1. 

Group 1 (*n* = 20): GP/AH Plus with continuous wave compaction.

Group 2 (*n* = 20): GP/BioRoot RCS with a single-cone technique. 

Group 3 (*n* = 20): GP/Endoseal MTA with a single-cone technique.

The AH Plus sealer was mixed in a 1:1 ratio according to the manufacturer’s instructions. The Endoseal MTA, a premixed sealer, was placed in a disposable syringe. One spoon of BioRoot RCS powder was mixed with five drops of a liquid consisting of water and calcium chloride. Each sealer was labeled with 0.1% rhodamine B dye (Sigma-Aldrich, St. Louis, MO, USA) during the mixing procedure to allow visualization under a confocal laser scanning microscope. In group 1, a size 40/0.06 GP cone (Dentsply Maillefer) was coated with the AH Plus sealer and placed into the canal. A heated System B plugger (SybronEndo, Orange, CA, USA) was used to remove the coronal part of the GP reaching into the apical 3 mm, and the remaining GP was compacted with an S-Kondenser (Obtura Spartan Endodontics, Algonquin, IL, USA). The middle and coronal areas were obturated vertically with the Obtura III Max System (Obtura Spartan Endodontics). The GP at the orifice level was compacted with an S-Kondenser (Obtura Spartan Endodontics). In groups 2 and 3, each sealer was applied to the root canal with a lentulo spiral (Dentsply Maillefer) to the working length, and a size 40/0.06 GP cone was inserted into the canal. Light pumping motions were applied to place the GP cone at the full working length. The GP at the upper surface was removed using a heated System B plugger (Kerr Cor., Orange, CA, USA) and compacted with an an S-Kondenser (Obtura Spartan Endodontics). The coronal access of all groups was sealed with a temporary filling material (Caviton; GC Corp., Tokyo, Japan). Finally, all experimental samples were stored for 7 days in wet conditions at 37 °C for complete setting.

### 2.3. Sample Sectioning

Each experimental sample was placed in a mold and embedded in self-curing resin, and then it was mounted and sectioned perpendicular to its long axis using a low-speed diamond wheel on a Metsaw (RB205 Metsaw-LS^TM^; R&B, Daejeon, Korea), which was set at 500 rpm with continuous water cooling. The horizontal cutting level was set at 3, 5, and 7 mm from the apex, and an approximately 1.0 ± 0.1 mm-thick section was collected.

### 2.4. Confocal Laser Scanning Microscopy Assay

All collected specimens were mounted onto a slide glass with a VECTASHIELD Antifade Mounting Medium (Vector Laboratories, Inc., Burlingame, CA, USA) and observed using CLSM (LSM 800 with Airyscan; Carl Zeiss Microscopy GmbH, Jena, Germany). To visualize and obtain accurate images, we observed and recorded the pictures using a 10× magnifying lens with size x: 512/y: 512 tiles. Several partial images were taken using a tile scan function and then assembled as a single full-specimen image using the stitching function in the ZEN imaging software (Carl Zeiss Microscopy GmbH). The excitation and emission wavelengths for rhodamine B were set at 558 and 575 nm, respectively. 

The deepest dentinal tubule penetration length of each sealer was calculated using ZEN lite 2012 (Carl Zeiss Microscopy GmbH, Jena, Germany). The mean fluorescence intensity (MFI) and sum fluorescence intensity (SFI) were automatically calculated by the ZEN lite software. The MFI and SFI correspond to the average and total intensity of the rhodamine B dye of selected pixels, respectively. The patterns of sealer penetration in dentinal tubules were evaluated by the ZEN lite 2.5D display and histogram processing. 

### 2.5. Statistical Analysis

The Kolmogorov–Smirnov normality test was used to verify the data distribution. Nonparametric tests were used because the data were not normally distributed. The results were distributed by experimental sealers and root levels. Kruskal–Wallis analysis was performed for overall comparisons. A series of Mann–Whitney *U* post hoc tests were used to compare the experimental sealers within the same root levels. The level of significance was set at *p*-values < 0.017, which were adjusted using the Bonferroni method.

## 3. Results

**Figure 1:**
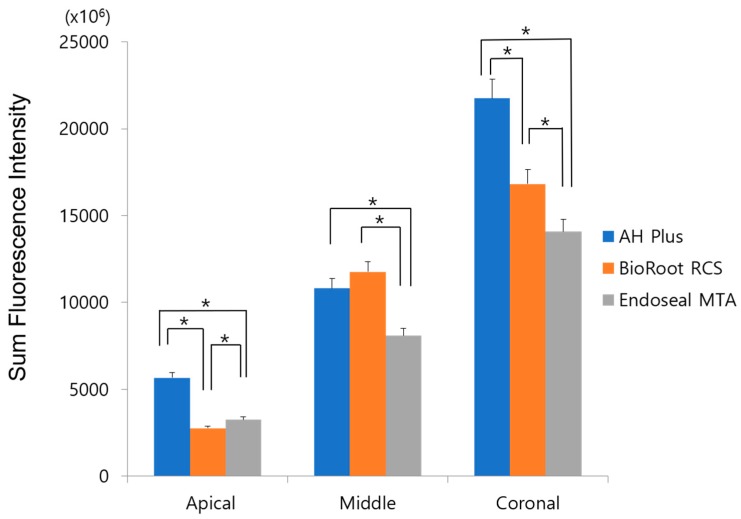
Sum fluorescence intensity of rhodamine B dye in the apical, middle, and coronal thirds of all experimental sealers. Stars indicate a statistically significant difference.

**Figure 2:**
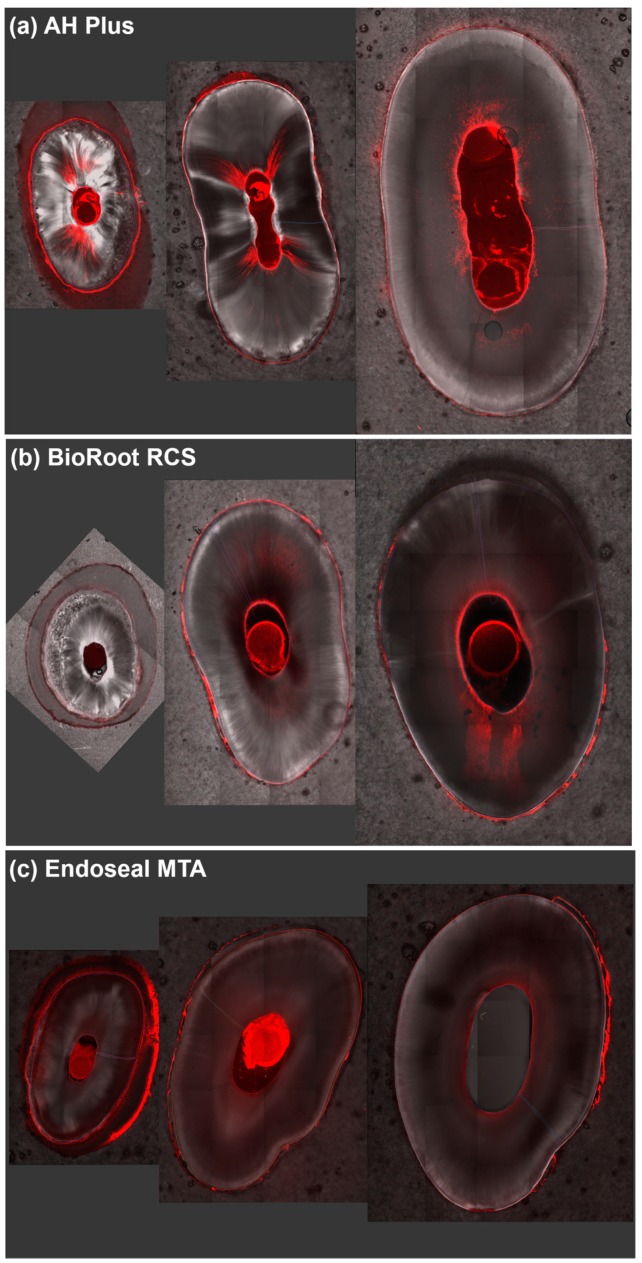
Representative images of the apical, middle, and coronal thirds: (**a**) AH Plus, (**b**) BioRoot RCS, and (**c**) Endoseal MTA.

Figure 1 shows the SFI of rhodamine B of all experimental groups at the apical, middle, and coronal levels. The highest SFI level was found in the coronal third and the lowest in the apical third in all experimental groups (*p* = 0.000). Among the experimental groups, AH Plus showed the highest intensity level in the apical and coronal thirds (*p* < 0.017); however, BioRoot RCS showed higher intensity in the middle area, similar to AH Plus (*p* > 0.017) (Figure 1). Representative images of each experimental group are shown in Figure 2. AH Plus showed a high intensity level of rhodamine B from the canal center into the dentinal tubules, whereas BioRoot RCS showed a relatively lower intensity in the broad dentinal tubule area. Endoseal MTA also showed sealer penetration in the broad dentinal tubule area; however, the intensity level was lower compared with those of AH Plus and BioRoot RCS.

**Figure 3:**
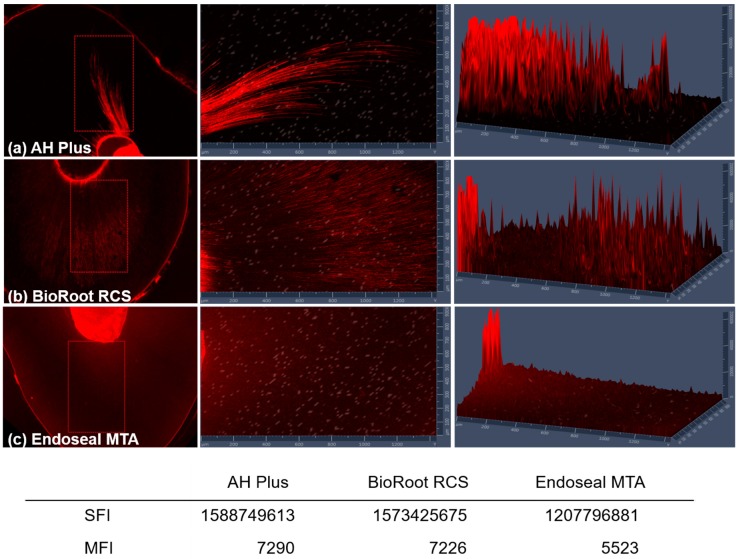
Representative image of a 2.5D histogram of each sealer at the middle third level: (**a**) AH Plus, (**b**) BioRoot RCS, and (**c**) Endoseal MTA. Abbreviations: SFI—sum fluorescence intensity and MFI—mean fluorescence intensity.

Using the ZEN software program, we reconstructed the penetration pattern of each sealer using 2.5D imagery of the middle third (Figure 3). AH Plus showed the highest intensity level of rhodamine B in the center of the root canal, and the intensity decreased gradually into the dentinal tubules (Figure 3a). By contrast, BioRoot RCS showed the highest intensity level in the center of the root canal; the intensity decreased into the dentinal tubules and increased again at a distance of 600–1200 μm from the root canal (Figure 3b). The SFI and MFI were almost the same for selected pixels of the AH Plus and BioRoot RCS groups. Endoseal MTA exhibited a relatively lower intensity just beneath the canal center (Figure 3c); additionally, its SFI and MFI were lower than those of AH Plus and BioRoot RCS. 

**Figure 4:**
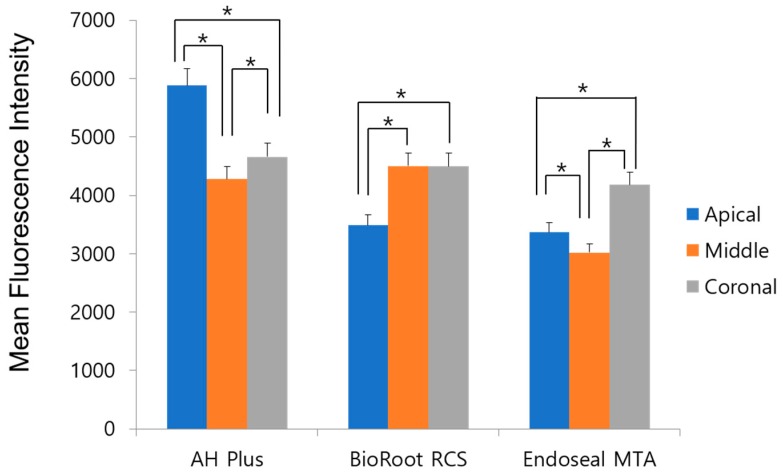
Mean fluorescence intensity of rhodamine B in all experimental sealers. Stars indicate a statistically significant difference.

Figure 4 shows the MFI of rhodamine B of all experimental groups at the apical, middle, and coronal levels. AH Plus showed a higher MFI in the apical area than in the middle and coronal areas (*p* < 0.017). In BioRoot RCS, the middle and coronal thirds showed a higher MFI than the apical area (*p* < 0.017); and in Endoseal MTA, the coronal third showed a higher MFI than the middle and apical thirds (*p* < 0.017). 

**Figure 5:**
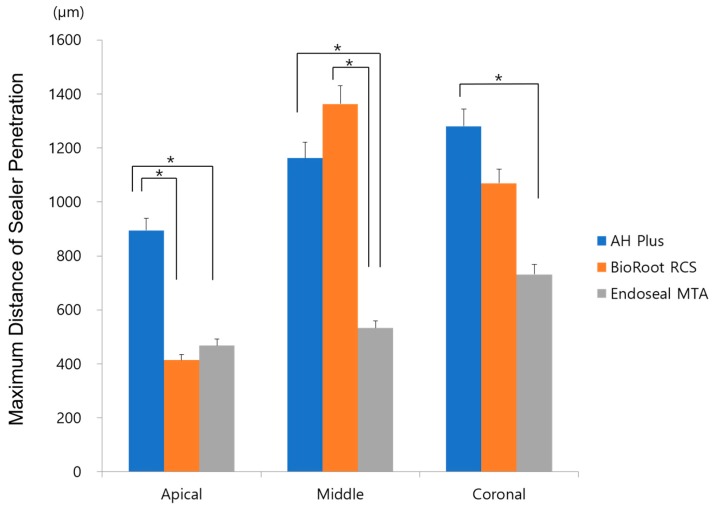
Maximum sealer penetration depth of all experimental sealers in the apical, middle, and coronal thirds. Stars indicate a statistically significant difference.

**Figure 6:**
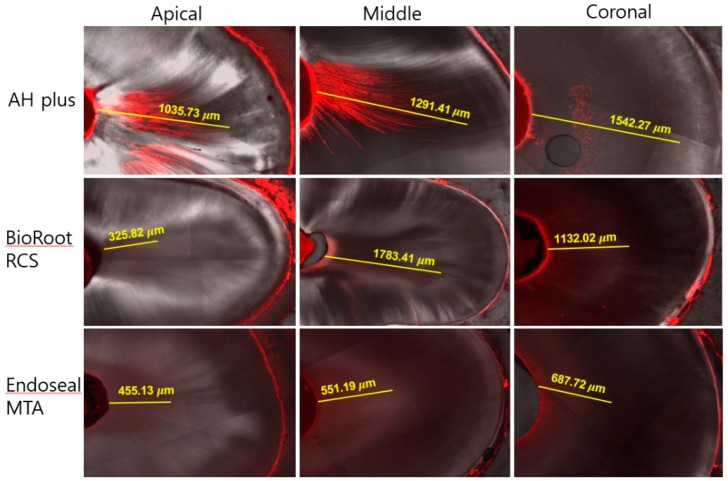
Representative image of the maximum sealer penetration of all experimental sealers in the apical, middle, and coronal thirds.

Figure 5 shows the maximum distance of sealer penetration. The AH Plus group showed higher sealer penetration at the apical third compared with BioRoot RCS and Endoseal MTA (*p* < 0.017), whereas BioRoot RCS showed higher sealer penetration in the middle and coronal thirds, similar to AH Plus (*p >* 0.017). Representative photos of the sealer penetration of each experimental group are shown in Figure 6.

## 4. Discussion

The purpose of this study was to compare the penetration ability of calcium silicate root canal sealers and conventional resin-based sealer using CLSM. The AH Plus group showed higher SFI in the apical and coronal thirds compared with those of BioRoot RCS and Endoseal MTA, whereas the BioRoot RCS group showed a higher intensity level in the middle third, similar to AH Plus. The maximum sealer penetration distance was higher at the apical third in the AH Plus group compared with BioRoot RCS and Endoseal MTA, and similar sealer penetration was observed at the middle and coronal third when comparing between AH Plus and BioRoot RCS.

BioRoot RCS has gained in popularity, as it shows fewer toxic effects on human periodontal ligament cells and induces osteogenic growth factor secretion [8]; additionally, cells in the BioRoot RCS extract spread better than those in the AH Plus extract [16], and its particles create mineral plugs through interactions with dentinal fluids [17,18]. The biomineralization ability of MTA has been reported in previous studies; this process is important for entombing intratubular bacteria and minimizing leakage [19,20]. This ability is also related to the enhanced bond strength. The development of a ‘mineral infiltration zone’ in the material in contact with the tissues indicates the formation of tag-like microstructures [21]. In our study, BioRoot RCS showed similar dentinal tubule penetration ability to AH Plus in the middle third. This represents an advantage of this sealer, as vertical condensation pressure is not necessary for obturation. 

In a previous study, BioRoot RCS showed higher calcium ion release than other sealers over a prolonged duration [18,22]. The prolonged mineralizing ion release triggers the nucleation of calcium phosphate, which may improve the sealing ability of obturation materials. In another study, a white precipitate was visible when BioRoot RCS specimens were immersed in phosphate-buffered saline [23]; notably, calcium hydroxide formation was observed earlier in the setting process.

In the study of Uzunoglu-Özyürek et al., BioRoot RCS provided higher dentinal tubule penetration than AH 26, even in the presence of calcium hydroxide [24]; however, in their study, canal fillings of all experimental groups were performed with a single GP cone combined with one sealer. This was the main difference from the method used in our study, in which AH Plus showed the deepest sealer penetration depth and higher intensity level in the apical third compared with BioRoot RCS; this is attributable to the continuous wave technique. We used System B up to the apical 3-mm level, thus providing a more complete apical seal for the AH Plus group.

Celikten et al. reported in a microcomputed tomography study that bioceramic sealers, such as EndoSequence BC (Brasseler USA, Savannah, GA, USA) and Smartpaste Bio (DRFP Ltd., Stamford, UK), produce voids; these results are similar to the findings for AH Plus [25]. A limitation of their study was that they used a single-cone filling technique in the AH Plus group. Zhang et al. used a fluid transport method to determine that there were no differences in sealing ability between iRoot SP (Innovative BioCeramix, Inc., Vancouver, Canada) using the single-cone technique and AH Plus using the continuous wave condensation technique [26]. They postulated that the reason could be that calcium silicate sealers, such as iRoot SP, do not shrink during setting and harden in the presence of water. However, iRoot SP with a single-cone technique showed higher leakage at all times for up to 8 weeks compared with both iRoot SP and AH Plus with continuous wave condensation, although the difference was not statistically significant [26]. 

Endoseal MTA has also demonstrated intratubular biomineralization, similar to that by BioRoot RCS. In a previous study, a finely pulverized pozzolan-based MTA sealer with a mean particle size of 1.5 µm, which was smaller than that of white ProRoot MTA, could infiltrate dentinal tubules [27]. Sealer tags and apatite precursors formed, allowing further propagation of crystallization [28]. 

In our study, Endoseal MTA showed a lower SFI in the apical, middle, and coronal thirds than AH Plus; maximum sealer penetration depths at all root levels were significantly lower than those of AH Plus. The reason Endoseal MTA showed relatively lower penetration into dentinal tubules was thought to be because we did not use ultrasonic activation during the filling procedures. Hwang et al. reported that Endoseal MTA with GP using ultrasonic vibration showed better sealer distribution than the AH Plus sealer with GP using a continuous wave technique [29]; they emphasized that the increase in the flowability of Endoseal MTA caused by ultrasonic vibration makes the canal obturation procedure easier and the filling of complex anatomical variations more complete. Kim et al. delivered ultrasonic power to Endoseal MTA through a GP cone and obtained a better filling quality with fewer voids [14]; from these results, they concluded that Endoseal MTA with ultrasonic activation provides better sealing ability.

MTA crystals are composed of calcium silicate hydrate. If MTA is exposed to an acidic environment, the cubic crystal begins to erode, significantly weakening the microhardness of the MTA [30]. In a previous study, Endoseal MTA exhibited more aggregated structures than those present in an EndoSequence BC sealer (Brasseler USA) and a Well-Root ST sealer (Vericom, Chuncheon, Korea), which showed more crystal-like structures [27]. In the same study, no Endoseal MTA exhibited crystallized structures, and the microhardness of the Endoseal MTA was relatively weaker than those of EndoSequence BC sealer and Well-Root ST [27]. Most calcium silicate sealers show higher solubility and dimensional change after immersion in water compared with resin-based conventional sealers [31]; therefore, their clinical application in open apex teeth should be performed carefully.

In this study, the CLSM method was used to assess sealer penetration into dentinal tubules, as done in previous studies [32,33]. CLSM offers several advantages over other techniques, as it does not require any special sample preparation and retains the integrity of the sealer. CLSM also provides the means to visualize the full extent of sealer penetration, as well as the sealer penetration depth [34]. Image acquisition from several optical sections using CLSM, even from thick specimens, allows for a more complete final image reconstruction [35]. 

## 5. Conclusions

In conclusion, the highest SFI level was found in the coronal third, and the lowest in the apical third in all experimental groups. AH Plus showed the highest SFI in apical and coronal areas, whereas the BioRoot RCS group showed a relatively higher intensity level in the middle area, similar to AH Plus. The maximum sealer penetration distance was higher at the apical third in the AH Plus group compared with BioRoot RCS and Endoseal MTA, and similar sealer penetration was observed at the middle and coronal third when comparing between AH Plus and BioRoot RCS.

## Figures and Tables

**Table 1:** Manufacturers and chemical compositions of experimental sealers.

Table	Manufacturer	Composition	Batch Number
AH Plus	Dentsply DeTrey GmbH, Konstanz, Germany	Epoxide paste: diepoxide, calcium tungstate, zirconium oxide, aerosil, and pigment; amine paste: 1-adamantane amine, *N*,*N*′-dibenzyl-5-oxa-nonandiamine-1,9, TCD-diamine, calcium tungstate, zirconium oxide, aerosil, and silicon oil	1703000226
BioRoot RCS	Septodont, Saint-Maur-des-Fossés Cedex, France	Tricalcium silicate, zirconium oxide (opacifier), and excipients in powder form, and calcium chloride and excipients as an aqueous liquid	B16422
Endoseal MTA	Maruchi, Wonju, Korea	Calcium silicates, calcium aluminates, calcium aluminoferrite, calcium sulfates, radiopacifier, and thickening agents	CD180327D
